# Particle Shedding from Cotton and Cotton-Polyester Fabrics in the Dry State and in Washes

**DOI:** 10.3390/polym15153201

**Published:** 2023-07-27

**Authors:** Tanja Pušić, Branka Vojnović, Sandra Flinčec Grgac, Mirjana Čurlin, Rajna Malinar

**Affiliations:** 1 Faculty of Textile Technology, University of Zagreb, 10000 Zagreb, Croatia; tanja.pusic@ttf.unizg.hr (T.P.); branka.vojnovic@ttf.unizg.hr (B.V.); rajna.malinar@ttf.unizg.hr (R.M.); 2 Faculty of Food Technology and Biotechnology, University of Zagreb, 10000 Zagreb, Croatia; mcurlin@pbf.hr

**Keywords:** cotton, cotton-polyester, washing process, particles, release, dry and wet state

## Abstract

The influence of 3, 10 and 50 washing cycles on the properties of cotton fabric and cotton-polyester blend in plain weave, was investigated in this study. In addition to the analysis of tensile properties in weft and warp directions and thickness, the number of particles produced in the dry state was also measured after 3, 10 and 50 washes. After washing, the entire effluent was analysed by determining the total suspended solids (TSS), the total solids (TS), the pH value and the conductivity. To determine the similarity of the observed wash cycles and properties of all processed samples, hierarchical cluster analysis (HCA) was performed. The fabric changes indicated by total wear in the warp direction after 50 washing cycles compared to unwashed ones amounting to 41.2% for cotton and 30.9% for cotton-polyester blend, may be attributed to the synergy of washing factors and raw material composition. Cotton fabric produced significantly more particles than cotton-polyester fabric in the dry state after the examined washing cycles in all size categories. A smaller number of released particles are in the larger size category >25 μm. The obtained TSS values confirm the degree of loading of the effluent with particulate matter from the analysed fabrics, since the detergent consists of water-soluble components. The HCA dendrograms confirmed that the release of particles during the first washing cycles is mainly determined by the structural properties of fabrics, while in the subsequent cycles the synergistic effect of chemical, mechanical and thermal effects in the interaction with the material prevailed.

## 1. Introduction

Environmental protection and methods to analyse the impact of textiles on quality and human health have been mandatory for many years and are now legal norms that need to be clarified through extensive product monitoring, such as circular economy principles. The choice of fibre is significant for the appearance of garments [1] and the environmental impact in the dry state and after washes. Previous research described textile dust released into the atmosphere as a result of the production and use of various types of textiles. Exposure to textile dust occurs during the production process, finishing, use, textile care and textile recycling. A major problem with the presence of textile dust is the possibility of various diseases arising from daily exposure. Despite the focus on cotton dust, the same challenges exist in the processing, finishing, and application of a variety of natural and synthetic materials. Particles shed from synthetic textiles represent a bigger problem, as they are deposited and cannot be broken down for the most part, which further pollutes the environment and people [2].

During the washing process, textiles are exposed to chemicals and mechanical agitation, which can create changes after repeated cycles. The extent of changes depends on textile characteristics (construction parameters, polymerisation degree, swelling capacity) as well as Sinner factors (chemicals, temperature, time, mechanical agitation) [3].

Detergent formulations are often alkaline and contain bleach as oxidising agents, which can trigger the chemical damage of cellulose when combined with other elements. The loss of tensile properties in cotton textiles is strongly linked to the depolymerisation of cellulose [4,5]. Lower washing temperatures, high efficiency detergents, and low bath ratios are required for the washing process to be sustainable, resulting in external fibrillation and pigment particle migration [6]. Cotton textile fibrillation may be associated with hydrophilicity, swelling, and construction features, namely the presence of shorter fibres in the yarn. This can be due to abrasion of the textile material in a wet (washing baths) or dry environment (tumble drying) [7,8]. Friction and deformation are two of the most common causes of clothing damage caused by washing machines [9,10]. The drying process at high temperature may affect the formation of cracks, and increased fibrillation.

Polyester fibres are characterised by high strength, crease resistance and fast drying, while cotton fibres are characterised by high strength, hydrophilicity and comfort [11]. These two fibre groups account for more than 75% of global production, and thus the largest share of textile waste [12]. Polyester fibres are semi-crystalline [13], hydrophobic, do not swell in water and are not prone to certain degradation and fibrillation. The influence of abrasion under wet and dry conditions is related to the generation of pilling [14,15]. Cotton and polyester textiles have different dimensional stability in wet and dry environments, in addition to significant variances in characteristics.

Cotton fibres in the alkaline medium can swell radially and longitudinally during finishing, causing shrinkage and increasing the take-up and yarn cross-section. In fibres where the orientation of the cellulose chains is in the direction of the fibres, swelling is greater in the transverse direction than in the longitudinal direction [16]. Increasing the crimp of washed cotton fabrics increases the elongation [17].

Polyester materials may shrink or become damaged if exposed to a temperature higher than the temperature of thermal transition. It was found that recycled polyester sheds almost 2.3 times more microplastics in washing compared to virgin polyester, as the strength of the fibre is reduced due to thermal exposure and shear degradation during the recycling process [18].

Cotton and polyester blends can meet special requirements for functional products, such as feel, appearance, dimensional stability, easy care and sufficient comfort [14]. The blends offer a number of advantages over pure cotton materials, including the ability to wash at lower temperatures, to reduce deposit content and are less damaged after multiple wash cycles.

On the other hand, the presence of microplastic particles (MP) in natural and wastewater, sediment, soil, aquatic organisms, and air has been linked to textile sources [19,20,21,22,23,24].

According to [25], “microplastic means particles containing solid polymer, to which additives or other substances may have been added, and where 1% *w*/*w* of particles have all dimensions 0.1 nm–5 mm, or a length of 0.3 mm–15 mm and length to diameter ratio of >3”.

The fragmentation [26], degradation [27], ageing [28], washing [29], and drying [30] of synthetic textile products are all possible sources of MP particles in environment [24]. Such products used for wet and dry cleaning of surfaces (mops), isolated synthetic fibres from vacuuming and drying, and fibres that come loose from clothing during home washing are the most common sources of MP in the environment [31,32]. Washing is thought to be responsible for around 35% of synthetic fibres in the environment [15,33].

The qualitative and quantitative determination of MP emissions and other released substances is difficult due to the variability and complexity of sources, as well as the fact that particulate matter is a vector for the dispersion of other emissions with varying degrees of risk in a real system. Numerous methods for determining particles were applied, either directly for the characterisation of dispersed systems [20] or after separation methods [34]. The choice of one or more methods of particle identification and/or separation in wet and dry environments is determined by knowledge of the system in which the particles originate. Total suspended solids (TSS) were found to be one of the best parameters for assessing the degree of particle loading in effluents and their separation [31]. Various filters with different pore sizes can be used to determine this parameter, such as glass fibre filters [35], polyethersulphonic filters [36], cellulosic filters [37,38], polyamide, polycarbonate, metallic and aluminosilicate filters [39,40].

The purpose of this research was to examine cotton fabrics and cotton-polyester blends after three, ten, and fifty cycles by measuring the released particles in the dry state and in washes. Particles in the dry state were released by cyclic bending, so the particle sizes of the washed fabrics were measured separately. The distribution of particles released from cotton fabrics and fabrics made of a blend of cotton and polyester was observed both individually and as a combined system. The effluents of the washing process of the aforementioned fabrics were analysed by determining the physico-chemical parameters, TSS, TS (total solids), pH and conductivity to determine the degree of particle load.

At the same time, the effect of 3, 10 and 50 washing cycles on the thickness and strength, which are the structural properties of the investigated fabrics, were monitored. It is well known that Fourier transform infrared spectroscopy (FTIR) provides information about changes in the chemical structure and environment of polymeric materials, such as: the presence or absence of certain functional groups: shifts in the frequency of absorption bands and changes in the relative intensity of the bands; the appearance of new peaks due to modifications; and the monitoring of changes during the life cycle of the material [41]. In addition, attenuated total reflectance Fourier’ transform infrared spectroscopy (FTIR-ATR) is a non-destructive analytical method that does not require lengthy sample preparation and has an exceptional wave number accuracy of 0.01 cm^−1^, which allows for the determination of low concentrations of individual groups of compounds [42]. For this reason, FTIR-ATR was applied in this study to evaluate the influence of 3, 10, and 50 washing cycles on the physicochemical changes of the cellulose polymer and polyethylene terephthalate that make up the cotton and cotton-polyester fabrics.

The originality of the research carried out can be seen in the connection between the amount of particles released from the fabrics in the dry state and and after the washing process.

## 2. Materials and Methods

### 2.1. Materials

Fabrics of 100% cotton and cotton-polyester (50:50) fabric in plain weave were produced on a Picanol OMNIplus 800 loom (air-jet loom, width 190 cm) at the Čateks d.o.o. textile mill, Čakovec, Croatia. The fabrics prepared in this way were scoured and, bleached according to the factory’s recipes and purchased for research purposes.The mass per unit area of cotton-polyester fabric is 158.6 gm^−2^, while the cotton fabric has a mass per unit area of 160.8 gm^−2^. Cotton and cotton-polyester fabrics have a warp and weft density of 20 picks per cm, and the fineness of two-ply yarn 14.2 tex.

The washing of the 3.6 kg fabrics, which contain 2.7 kg of cotton and 0.9 kg of polyester (3:1), was carried out in accordance with HRN EN ISO 15797:2002 using a standard detergent with fluorescent whitening agent, the composition of which is shown in Table 1, to which 2 g/L peracetic acid (PAA) was added as a bleach in the washing process.

### 2.2. Washing Process

The cotton and cotton-polyester blend fabrics were washed in the laboratory washing machine Wascator FOM71 CLS by Electrolux at 75 °C using programme 2 with 3, 10, and 50 washing cycles. All process parameters comply with the standard, but due to the importance of the influence of the process conditions, a detailed description of programme 2 is included in Table 2.

After washing, the samples were air-dried. Table 3 shows the designations applied to samples before and after washing.

### 2.3. Methods

In order to monitor the influence of washing on the structural properties of fabrics before and after the washing cycle, the thickness was tested according to HRN EN ISO 5084:2003 Textiles -- Determination of thickness of textiles and textile products at ten different places using a thickness gauge DM 2000—Wolf, Germany with a precision of 0.001 mm. The thickness gauge is made up of two parts: a support to hold the material in place and a device called a pressure plate, which is a 25 cm^2^ circular plate that presses down on the material at a specific pressure (preload of 0.5 kPa). Tensile properties were measured on the samples in the weft direction before and after the washing cycles according to EN ISO 13934-1:1999 Textiles—Tensile properties of fabrics—Part 1: Determination of maximum force and elongation at maximum force using the strip method on a TensoLab Strength Tester (Mesdan S.p.A., Puegnago del Garda, Italy), distance between clamps 100 mm, bursting speed 100 mm/min and pretension 2 N.

Determination of overall decrease in breaking strength (total wear) was calculated according to ISO 4312:1989: Surface active agents—Evaluation of certain effects of laundering—Methods of analysis and test for unsoiled cotton control cloth.

The total wear, *U*_*t*_ of the fabrics was calculated according to Equation (1):

(1) $$U_{t}=\frac{F_{0}-F}{F_{0}}\cdot 100$$

where *U*_*t*_ is total wear (%), *F*_0_ is breaking force of unwashed fabric (N), and *F* is breaking force of washed fabric (N).

Cotton and cotton-polyester fabric before and after 3, 10 and 50 washing cycles were analysed using Fourier transform infrared spectroscopy (FTIR, PerkinElmer, Spectrum 100, Shelton, CT, USA) with the attenuated total reflection (ATR) measurement technique obtained spectral curves were processed in Spectrum 100. Four scans were performed for each sample with a resolution of 4 cm^−1^ between 4000 cm^−1^ and 380 cm^−1^

The generation lint and other particles was measured on LasAir III (Particle Measuring Systems) laser particle counter connected to a particle generator in a laminar airflow booth. Samples were prepared according to EN ISO 9073-10 and mounted on the particle generator and subjected to controlled bending. The number of particles released during the test was measured in the following size categories: 0.3 µm for particle sizes of 0.3–0.5 µm; 0.5 µm for particle sizes of 0.5–1 µm; 1 µm for particle sizes of 1–5 µm; 5 µm for particle sizes of 5–10 µm; 10 µm for particle sizes of 10–25 µm; 25 µm for particle sizes larger than 25 µm. For each sample, the measurements were performed on 5 test tubes. This method was adopted from EN ISO 9073-10 Textiles—Test methods for nonwovens—Part 10: Lint and other particles generation in the dry state, with the test time adjusted to 30 min [2].

The entire effluent (hence referred to as effluent) collected from the washing procedure and three rinse cycles was analysed as part of the research. Methods for characterising effluent after 3, 10 and 50 washing cycles include selected physicochemical parameters such as TSS, TS, pH, and conductivity.

The total suspended solids (TSS) of effluents were determined by the standard gravimetric method. After membrane filtration of effluent using 0.7 µm fibre glass filter (GF), mass of GF with filter cake as residue after drying at 100 °C was determined.

The total solids (TS) of effluents were determined by the standard gravimetric method in evaporating dish at 105 °C until constant mass was achieved.

pH and conductivity of effluents were determined using pH meter, Schott, ProLab 3000 and conductometer, CG 853, Schott, respectively.

To determine the similarity of the observed wash cycles and similar properties of all processed samples, hierarchical cluster analysis (HCA) was performed using a Minitab software, and graphs in the form of Ward’s dendrograms showed the homogeneous groups or clusters whose variables are connected by a certain similarity [43].

## 3. Results

The effect of the cyclic washing procedure on the qualities of cotton fabrics and cotton-polyester blends was studied by monitoring structural features, thickness, and tensile properties. These fabrics were washed under the same conditions, and it is predicted that the percentage of cotton component will have an effect on thickness variations during washing cycles.

### 3.1. Structural Parameters

The average values and coefficients of variation of the measured fabric thicknesses before and after 3, 10, and 50 washing cycles are presented in Table 4.

The data in Table 4 show that the cotton fabric has a larger thickness before washing than the unwashed fabric consisting of a cotton-polyester blend. Due to the influence of the parameters of the washing process, the thickness of the cotton fabric increases. Statistical results indicate that cotton textiles shrink unevenly when washed. The cotton-polyester blend showed the highest difference in fabric thickness after three cycles as compared to unwashed material. Further washing cycles had a significantly lower influence on the thickness change, indicating no further scatter in relation to the structural features of the sample in terms of the coefficient of variation. The swelling of cotton fibres in the alkaline medium of the washing bath causes shrinkage, which modifies the cross-section of the yarn and results in differences in fabric thickness. The hydrophobic polyester component of the blend is more resistant to all washing parameters and guarantees dimensional stability of the fabric.

Table 5 groups the average values of breaking strength and elongation of cotton and cotton-polyester samples before and after the third, tenth, and fifth washing cycles.

The breaking forces of unwashed cotton fabrics and cotton-polyester blend fabrics differed in the warp and weft directions, and the value in the warp direction was twice that of the weft direction. The breaking force of the examined fabrics (Table 5) in the warp direction exceeds 1000 N, indicating a high degree of structural integration. In contrast to the consolidation of the fabric in the warp direction, the breaking force in the weft direction is modest and is roughly 480 N for both fabrics.

Total wear (Ut) was calculated in the direction of the warp and weft throughout the washing process, which included tensile changes caused by mechanical and chemical degradation of the washed sample in relation to the unwashed sample, Table 6.

The results for the breaking forces and calculated Ut (Table 5 and Table 6) show that there is a decrease in breaking strength, i.e., an increase in damage (total wear) after 3, 10, and 50 washing cycles in the direction of the warp threads for both fabrics tested (cotton and cotton-polyester blend). The increase in strength of the cotton-polyester fabric after 3 and 10 washing cycles was recorded in the weft direction. Structural properties, fibrillation and shrinking of materials during the washing process are the contributing factors to this relationship. The total wear of the cotton fabric (Table 6) after 50 washing cycles in the warp direction was 41.19%, which was higher than the 30.98% of the cotton-polyester blend fabric. The resulting damage can be attributed to a synergistic effect, including the raw material composition, the degree of soiling of the fabrics, and all components of the Sinner’s circle. It is in line with the study of oxidation and alkali promoted reactions on cotton cellulose [4]. Standard alkaline detergent with peracetic acid (PAA) as an oxidising agent at 75 °C acts on the fibrillation and depolymerisation of cotton cellulose, as well as the high alkalinity of the polyester component of the blend. However, total wear in the weft direction of almost all washed fabrics has a negative sign, indicating that no damage has been caused by the wash. Given the predicted equivalent influence of significant washing process parameters on the fabrics in the direction of the weft threads, these values may be explained by fabric shrinkage during the washing process. Based on the results presented, the impact of the washing cycle on the cotton component is more pronounced under the applied conditions.

Standard detergent does not include the enzyme cellulase, which eliminates cellulose fibrils during the washing process [44]. When textiles are dried in a stationary ambient atmosphere, the migrating fibrils remain on the surface [45] and tend to loosen in both dry and wet conditions.

### 3.2. Physicochemical Analysis of Samples with FTIR-ATR

**Figure 1:**
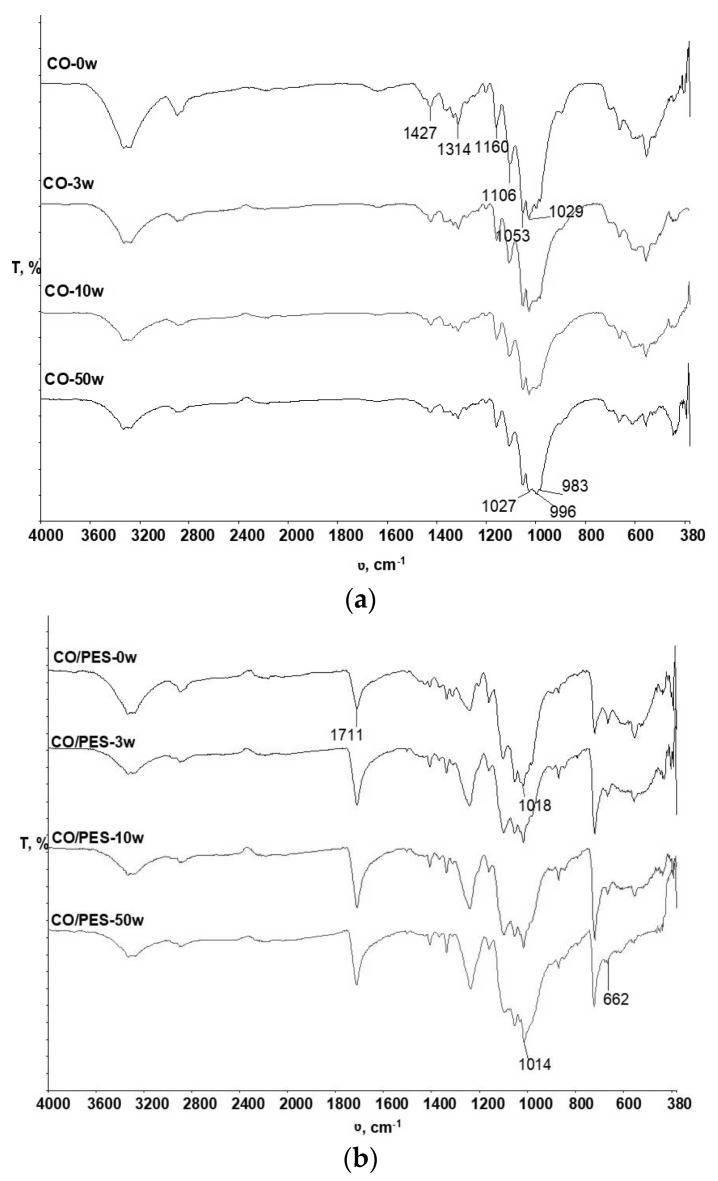
FTIR spectra of the cotton (**a**) and cotton-polyester (**b**) samples before and after 3, 10, and 50 wash cycles.

The spectral curves of the cotton and cotton-polyester fabrics before and after 3, 10, and 50 wash cycles are showen in Figure 1.

From Figure 1a,b, no significant changes were observed in the spectral bands obtained for the cotton and cotton-polyester fabric samples (CO-0w and CO /PES-0w) before and after 3 and 10 washing cyclesn (CO-3w, CO-10w and CO/PES-3w, CO/PES-10w), respectively. The spectral band of the sample CO-50w shows changes in the shape and intensity of the peaks in the range of 1027 cm^−1^, where the peak is due to the vibration of the C-OH bond of primary alcohols, and in the ranges of wavenumbers 996 cm^−1^ and 983 cm^−1^, where the peaks indicate vibrations within the—CH—bond, indicating that minor changes occurred within the polymer as a result of the washing process under the influence of chemistry as described. Minor changes can also be seen in the CO/PES-50w sample, specifically by the increase in the intensity of the peak at wavenumber 1014 cm^−1^, indicating an enhancement of the vibrations in the ester group (O=C-O-C) of the polyester component, which also indicates a higher surface wear of the cotton component within the CO/PES blend, which was also confirmed by the analytical methods in the previous research [46,47].

### 3.3. Particle Shedding from Fabrics in Dry State

The number of particles thus produced in the dry state was determined by applying cyclic bending. In view of the material differences mentioned above, the test was carried out for cotton and cotton-polyester fabrics. The obtained results refer to the release of lint and other particles from the material due to the deposits formed during the washing process.

Based on the number of particles released in Table 7, a minor proportion of the particles released are in the larger size category >25 µm. During the washing cycles analysed, significantly more particles in all size categories were released from the cotton fabric compared to the cotton-polyester fabric.

**Figure 2:**
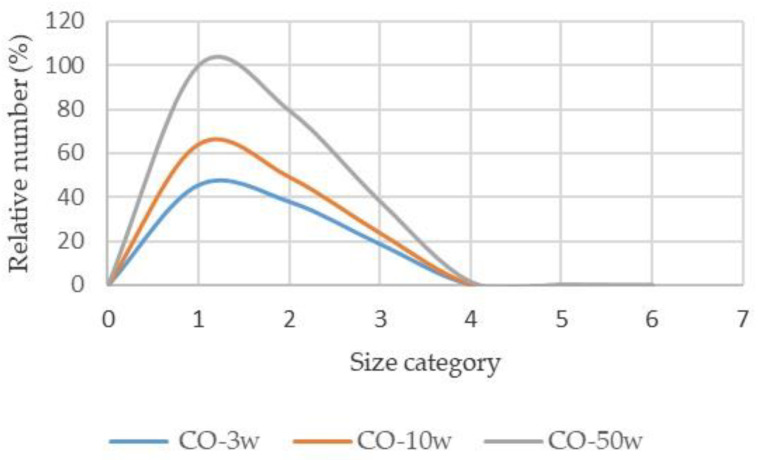
Relative number of particles released from cotton fabric according to size category (1–6).

**Figure 3:**
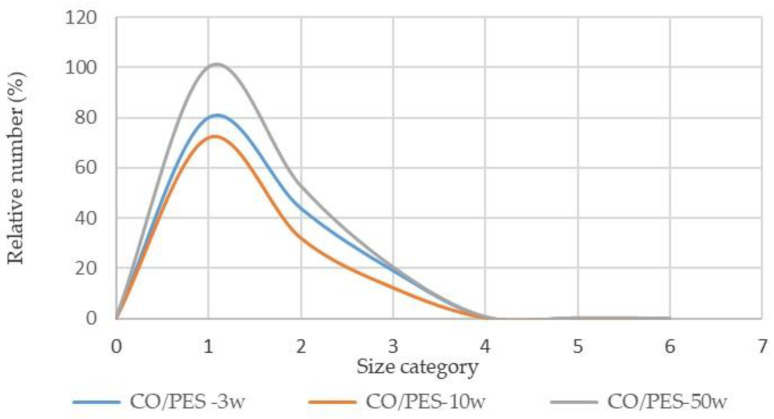
Relative number of particles released from cotton-polyester fabric according to size category (1–6).

The relative values of the number of particles for each material were determined in proportion to the largest number of released particles in order to compare the results based on the washing cycles. The results are shown in Figure 2 and Figure 3 with the following size categories: 0.3 µm for particle sizes of 0.3–0.5 µm (1); 0.5 µm for particle sizes of 0.5–1 µm (2); 1 µm for particle sizes of 1–5 µm (3); 5 µm for particle sizes of 5–10 µm (4); 10 µm for particle sizes of 10–25 µm (5); 25 µm for particle sizes larger than 25 µm (6).

According to the graph shown in Figure 2 and Figure 3, the smaller particle size category in both materials fluctuates more depending on the wash cycle. The number of wash cycles has a different effect on the number of particles released from different materials. For cotton fabrics, the correlation between the number of washing cycles and particle size in the first four categories is higher as the number of washing cycles increases, but the number of washing cycles has a different effect on cotton-polyester fabrics. There are more particles between 0.3 and 5 µm in size than particles larger than 5 µm. These results also reflect the thermal, chemical, and mechanical effects of the washing cycles on the fibre. Standard program 2 was used to wash cotton fabrics and cotton-polyester blended fabrics. This program required the addition of a chemical bleach, PAA, in addition to a standard detergent. Cotton textiles become more fibrillated as the number of washing cycles rises [7], but in this system, due to the lack of soiling, peracetic acid in alkaline at 75 °C may affect cotton cellulose depolymerisation and cellulose fibril formation.

### 3.4. Particle Shedding from Fabrics in Wash Cycles

The effluent after washing can be contaminated by soil from textiles, lint, dyes, finishing agents, and detergents [48]. Some detergents contain insoluble components that pollute the effluent and at the same time obscure the filter along with the MP particles, making microscopic analysis of the released MP particles difficult. Therefore, washing synthetic textiles in water is carried out in some studies [49].

Since cotton and cotton-polyester fabrics in the study have not been finished, dyed and soiled, the effluent can be contaminated with particles of textile origin and detergent. The components of the used standard detergent are soluble, so contamination by particles originating from the detergent is not expected. It can therefore be assumed that the contamination of the wash effluent contains particles from cotton and cotton-polyester fabrics. Effluent characteristics after 3, 10 and 50 washes of cotton and cotton-polyester fabrics are presented in Table 8.

The high pH value of the studied effluents is nearly consistent during all washing cycles and is caused by the alkaline components in the detergent. This characteristic is not expected to change despite the dilution that occurs during the three-step washing process. In this procedure for washing textiles without soiling [50], the alkalis could not be used to saponify grease and neutralise acidic soiling, but they functioned synergistically with anionic surfactants and bleach (PAA). A high alkali concentration can also be hazardous to particular textiles, such as damaged cotton and PES textiles, which get hydrolysed [51] as a result, causing wear and tear and shortening the product’s life.

The conductivity of the effluents under consideration given in Table 8 reflects the quantity of detergent components that are water soluble. Again, the washing process was performed on fabrics with no soiling, implying that the detergent ingredients were not focused on their removal but on potential interactions with the fabrics, causing structural changes and a loss of fabric integrity in the direction of the warp threads.

Based on this, it is clear that the composition of the reference detergent with the addition of PAA and other parameters of the Sinner cycle (temperature, mechanics, time) influence the polymeric structures of cotton cellulose and cotton-polyester during the washing cycles. The TSS values obtained, as reported in Table 8, confirm the degree of particulate load in the effluent. Given the composition of the detergent in Table 1, which consists exclusively of water-soluble components, it is probable that the TSS consists of particles released by the studied textile materials. Although the relationship and type of the particles were not determined in this study, earlier studies on the effect of washing on the release of particulate matter from cotton-polyester blend fabrics have shown that the filter cake contains fibrillary particulate matter of both polymer substances [52].

### 3.5. MVA-Similarities and Disimilarities of Observed Systems in Dry State and Washes

**Figure 4:**
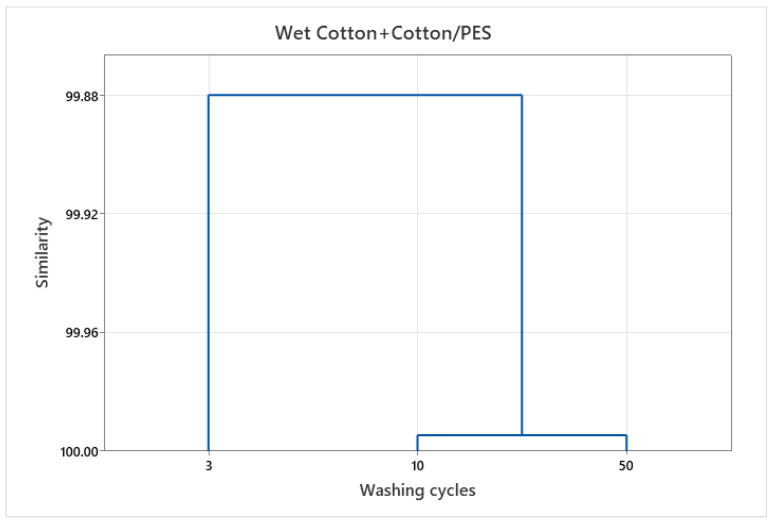
Dendrogram of the HCA according to Ward for similarities/dissimilarities of washing cycles for cotton and cotton-polyester in washes.

**Figure 5:**
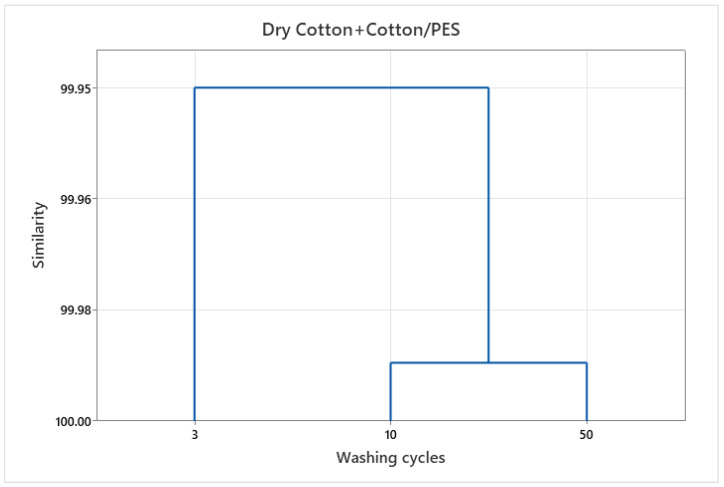
Dendrogram of the HCA according to Ward for similarities/dissimilarities of washed cotton and cotton-polyester fabrics in dry state.

**Figure 6:**
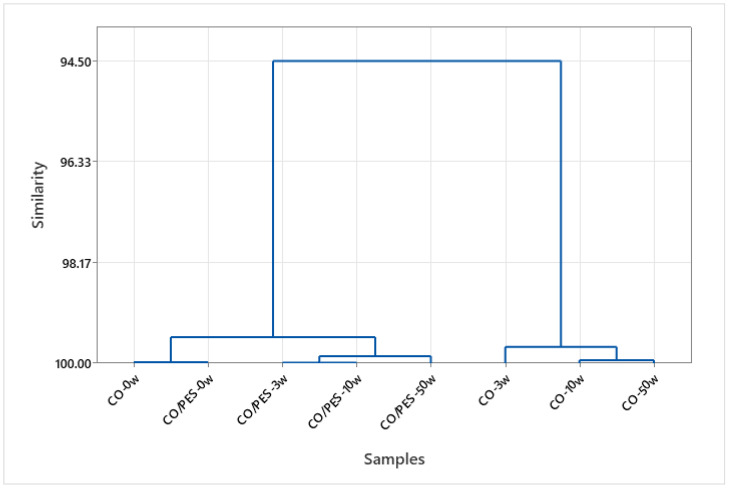
Dendrogram of the HCA according to Ward for similarities/dissimilarities of samples based on data set of tensile properties, total wear and thickness.

The different systems (wash-wet and dry) to which the cotton and cotton-polyester fabrics studied were exposed and the different physicochemical properties in each system required the use of advanced statistical methods. Multivariant analysis was used to determine system similarities in terms of the number of particles released from the examined fabrics in procedures with varying numbers of washing cycles. The results using the Ward dendrogram are shown in Figure 4, Figure 5 and Figure 6.

The different distribution of clustering with regard to the number of washing cycles indicates that cotton contributes to the increasing release of particles with a higher number of washing cycles, while the contribution of polyester is greater in a lower number of washing cycles.

The results include the formation of clusters, where a data set was selected for particle release from cotton and cotton-polyester fabrics in washes, Figure 4 and dry state, Figure 5 for different washing cycles. The clusters formed are very similar, but a separate cluster can be identified that includes effluents from 10 and 50 wash cycles, while the effluent from 3 washing cycles was separated.

The dendrograms of clustering also show a high degree of similarity, but the cluster distribution is different with respect to the number of washing cycles. The washing conditions, especially the high alkali content, affect the damage of the textiles, which contributes to the release of particles.

Tensile properties, thickness, and total wear in the weft and warp directions were examined to establish the importance of textile composition and washing cycles to particle release, Figure 6.

In the initial washing cycles, the structural properties of the material play a significant role in the release of particulate matter; however, in subsequent cycles, the interaction between the chemical, mechanical and thermal factors and of the fabrics becomes increasingly prominent.

## 4. Conclusions

This research examines the chemical, mechanical, and thermal impacts of 3 washing cycles, 10 washing cycles, and 50 washing cycles on 100% cotton and a 50/50 cotton-polyester blend in plain weave with no soiling in the dry state and after washes utilising fabric and effluent characteristics. Tensile properties, thickness and generated particles in the dry state depend on the number of washing cycles. Increased strength of cotton-polyesteru fabric after 3 and 10 washing cycles in the weft direction is the result of fibrillation and shrinkage. Changes in fabric properties, expressed as total wear in the warp direction after 50 washing cycles compared to unwashed amounting to 41.2% for cotton and 30.9% for cotton-polyester blend, can be attributed to the synergy of process parameters, fabric structure and raw material composition.

The number of particles released in the dry state > 25 µm is significantly lower than the number of particles released in the size of 0.3 to 5 µm. In all size categories, the quantity of particles released in the dry state is much larger from washed cotton fabric than from washed cotton-polyester fabric. Because the detergent contains water-soluble components, the TSS values obtained confirm the degree of contamination of the effluent with particles from the tested textile materials.

From the spectral bands of CO and CO /PES samples before and after 3, 10 and 50 washing cycles, no significant changes at the physicochemical level within the polymers of the tested samples were identified.

According to HCA dendrograms, particle release in early wash cycles is mostly regulated by material structure. Future wash cycles are influenced by chemical, mechanical, and thermal interactions. The findings of the research highlight the need for the use of an analytical approach to categorise and quantify particles released from textiles in both the dry state and washes in order to decrease the potential for detrimental impacts on humans and the environment.

## Figures and Tables

**Table 1:** Composition of reference detergent according to HRN EN ISO 15797.

Composition	Percentage
ABS-Na (C-12 chain)	0.425
Nonionic surfactant (C13/15 7EO or C12/14 7EO)	6.0
Sodium citrate dihydrate	5.0
Hydroxyethanediphosporic acid Na salt (HEDP)	1.0
Metasilicate anhydrous	42.3
Polymer (polymaleic acid)	2.0
Foam inhibitor (phosphoric acid ester)	3.0
Sodium carbonate	39.5
Fluorescent whitening agent	0.3
Water	0.475

**Table 2:** Washing procedure.

load ratio	1:17
agitation during heating, washing and rinsing	normal
**washing**	
liquor ratio	1:4
detergent additive	4 g/L detergent 2 g/L PAA
temperature	75 ± 2 °C
time	20 min
cool down	yes
drain	1 min
interspin	No
**rinse 1**	
liquor ratio	1:5
time	3 min
drain	1 min
interspin	1 min
**rinse 2**	
liquor ratio	1:5
time	3 min
drain	1 min
interspin	1 min
**rinse 3**	
liquor ratio	1:5
time	3 min
drain	1 min
final extraction	6 min
residual moisture	35–40%

**Table 3:** Designations of samples.

Designation	Sample
CO-0w	Cotton fabric before washing
CO-3w	Cotton fabric after 3 washing cycles
CO-10w	Cotton fabric after 10 washing cycles
CO-50w	Cotton fabric after 50 washing cycles
CO/PES-0w	Cotton-polyester fabric after 3 washing cycles
CO/PES-3w	Cotton-polyester fabric after 10 washing cycles
CO/PES-10w	Cotton-polyester fabric after 50 washing cycles
CO/PES-50w	Cotton-polyester fabric after 3 washing cycles

**Table 4:** Average value and standard deviation of the fabrics before and after the washing cycles.

Sample	Thickness (mm)	CV (%)
CO-0w	0.440	2.83
CO-3w	0.489	6.35
CO-10w	0.516	6.66
CO-50w	0.553	8.61
CO/PES-0w	0.384	2.20
CO/PES-3w	0.443	1.52
CO/PES-10w	0.443	1.52
CO/PES-50w	0.461	2.97

**Table 5:** Breaking strength (F) and elongation (Ɛ) of cotton and cotton-polyester fabrics in warp and weft directions.

Samples		F (N)	Ɛ (%)
CO-0w	weft	483.8	6.46
	warp	1028.8	8.94
CO-3w	weft	501.8	6.82
	warp	706.0	15.16
CO-10w	weft	582.2	7.84
	warp	729.8	18.00
CO-50w	weft	518.8	8.42
	warp	605.0	20.14
CO/PES-0w	weft	480.2	14.64
	warp	1074.6	16.85
CO/PES-3w	weft	541.6	14.25
	warp	1002.8	21.01
CO/PES-10w	weft	511.8	14.05
	warp	983.8	22.20
CO/PES-50w	weft	442.6	13.10
	warp	741.6	22.60

**Table 6:** Total wear (Ut) of fabrics in warp and weft directions.

Ut (%)						
		CO			CO/PES	
	3w	10w	50w	3w	10w	50w
weft	−3.721	−20.339	−7.234	−12.786	−6.581	7.830
warp	31.376	29.063	41.194	6.6816	8.450	30.988

**Table 7:** Average number of particles released in each category.

		Number of Released Particles					
Fabric	Cycles	0.3–0.5 µm	0.5–1 µm	1–5 µm	5–10 µm	10–25 µm	≥25 µm
CO	3w	1,972,940.2	1,642,870.4	808,332.4	16,146.0	3305.8	745.8
	10w	2,782,562.8	2,133,212.3	1,030,349.3	28,086.5	4983.0	628.8
	50w	4,347,945.4	3,452,533.2	1,655,430.8	65,939.8	12,165.2	744.8
CO/PES	3w	51,192.0	28,188.6	12,296.4	347.0	120.6	80.4
	10w	45,924.0	20,549.2	7887.8	233.0	82.0	47.4
	50w	63,962.8	33,833.4	13,122.0	428.8	116.2	60.4

**Table 8:** Physicochemical properties of effluent from washing cotton and cotton-polyester fabrics.

	Washing Effluent		
Parameter	3w	10w	50w
pH	9.94	9.99	9.89
Conductivity (µS/cm)	1859	1927	1782
TSS (mg/L)	117	77	81
TS (mg/L)	3394	3452	3298

## Data Availability

Not applicable.
